# Enhancing Diagnosis and Prognosis by Assessing the Clinical, Morphological, and Behavior Aspects in Soft Tissue Sarcomas

**DOI:** 10.7759/cureus.64025

**Published:** 2024-07-07

**Authors:** Bogdan Serban, Mihnea Ioan Gabriel Popa, Adrian Cursaru, Bogdan Cretu, Georgian L Iacobescu, Catalin Cirstoiu, Sergiu Iordache

**Affiliations:** 1 Orthopedics and Traumatology Department, University Emergency Hospital, Bucharest, ROU; 2 Orthopedics and Traumatology Department, Carol Davila University of Medicine and Pharmacy, Bucharest, ROU

**Keywords:** soft-tissue sarcomas, risk factors, immunohistochemistry, histopathology, targeted therapies, risk stratification, malignancies

## Abstract

Soft-tissue sarcomas (STSs) are an uncommon and diverse group of cancers, consisting of more than 80 different kinds, each showing unique mesenchymal differentiation as described by the World Health Organization (WHO). The prognostic factors at the time of diagnosis mostly depend on the size, depth, and histological grade of the lymphatic involvement. Improved prognostic indicators are necessary to identify patients at high risk who may derive advantages from adjuvant therapy and those at low risk who might avoid treatment-related side effects. Over a period of five years, a considerable number of patients experience the recurrence of the tumor in the same area or the metastasis of the disease to other parts of the body, even after the complete removal of the localized tumor through surgery. To further personalize and focus medicines, it is critical to enhance prediction accuracy and uncover new therapy targets. This is particularly important considering the high mortality and morbidity rate associated with metastatic STS. The significant diversity of STS poses difficulty in comprehending its pathobiology and in converting biological progress into therapeutic application.

This prospective cohort study was carried out at a major university hospital to ascertain adult patients who were diagnosed with STS of the extremities between the period from 2018 to 2023. The inclusion criteria were individuals who were 18 years of age or older with a histological diagnosis of STS. The exclusion criteria encompassed individuals with malignancies other than STS and those with inadequate data on essential factors. Thorough assessments were conducted to analyze patient demographics, tumor features (including site, size, depth, neurovascular or bone invasion), and histologic type and grade (according to the French Federation of Cancer Centers (FNCLCC) grading system). The purpose was to find predictive markers and evaluate results.

The results are consistent with previous research and enhance our current knowledge of STS prognosis. Key prognostic markers for metastasis and mortality risk include tumor size larger than 5 cm, histologic grade, and sarcoma subtype. Radical surgical procedures, such as amputation or disarticulation, did not show any survival advantage, probably because they were used in situations where the disease had already progressed locally and had significant involvement in the blood vessels. Histologic grading has been identified as the most important factor in predicting the likelihood of metastasis in adult STSs. The study found that most tumors were of high grade, and there was a statistically significant association between tumor grade, Ki67 levels, and overall survival. A small proportion of patients experienced prolonged longevity beyond five years, emphasizing the connection between early detection, tumors of lesser severity, and enhanced results. These observations emphasize the significance of accurate prognostic assessments and customized therapeutic approaches in the treatment of STS.

## Introduction

Among the uncommon and diverse tumors with more than 80 histological subgroups, soft-tissue sarcomas (STSs) show varied mesenchymal differentiation. They exhibit a broad range of histological subtypes and clinical manifestations. These tumors provide particular difficulties in diagnosis, treatment, and prognosis due to their potential for aggressive growth, metastasis, and unpredictable response to therapy [1,2].

The diagnosis and clinical management of STS is difficult due to the nonspecificity of its clinical manifestations and its rarity, especially in non-dedicated sarcoma centers. Patients usually endure an extended period between presentation and a confirmed diagnosis, and they could be dealing already with advanced disease [3]. Although the five-year overall survival (OS) rate for STS is 55%-65%, OS might drop to 15% if distant metastases are present [4]. Within five years, 40%-50% of individuals experience locally recurrent or metastatic disease, even after having their localized disease completely surgically removed [1]. However, distant metastasis and local recurrence rates vary greatly depending on the histological subtype and anatomical site [5].

Clinical, pathologic, and biologic variables engage intricately to affect the patient prognosis and biologic behavior of STSs. The vast variability of these cancers, their comparatively low incidence, and their distinctive and unpredictable behavior pose challenges when looking into prognostic factors. Numerous research studies on STSs address the significance of different demographic, clinical, and therapy characteristics in terms of prognosis. Numerous prognostic markers, including local recurrence, metastasis-free survival (MFS), post-metastasis survival, and OS, have been identified to have statistical significance by univariate analysis for various outcomes. It is still debatable whether a consistent set of prognostic indicators has been documented, with the exception of certain variables like size, depth, and tumor grade. In addition, there was insufficient statistical power to examine parameters including anatomic site and histopathologic subtypes [6]. The most crucial component of STS staging is grade, but even so, there is an ongoing debate in cancer research on this parameter as well as a number of other variables that affect patient prognosis, including age, sex, and margins [7]. Establishing the prognosis of the tumor and discovering novel therapeutic targets is essential in order to facilitate specified individualized treatment approaches since metastatic STS has an elevated mortality rate in order to effectively manage this type of pathology and improve the patient outcome. Searching for tumor characteristics that exhibit a prognostic value for metastasis development turned up a plethora of independent factors, as one might have anticipated. The tumor's local behavior was linked to several of these, while its histomorphology was connected to many of them [8].

Risk stratification in STS

The clinical management of STS nowadays is intricate and frequently poorly defined. STS surgery involves a significant chance of morbidity especially in older patients or when implemented for large tumors in intricate anatomical locations. Moreover, chemotherapies and many of the targeted therapies in development have significant associated toxicities, for which many patients see little to no benefit. By using risk stratification, it is possible to estimate the probability that a patient will encounter negative outcomes, such as medication side effects or disease progression (metastasis, recurrence, or death). The possible efficacy of an intervention, a patient's psychological and social well-being, and medical risk stratification must all be carefully assessed. However, risk stratification can help guide therapy pathway decisions, improve patient-clinician communication, and more if properly used [2,5,6].

Clinicopathological information, comprising tumor grade, size, and depth, is consistently documented for every patient and helps clinicians assess the degree of disease progression. The understanding of clinical conditions guides interventional decision-making and serves as an informal risk assessment for occurrences associated with the disease, even though formal risk stratification is not provided by this. Clinicopathological factors in STS have been evaluated for their predictive significance through extensive retrospective investigations. The anatomical site and histological subtype are additional significant determinants of outcome. Distinct STS subtypes exhibit varying inclinations towards metastasis and local recurrence. Retrospective data show that the MFS and local recurrence-free survival (LRFS) of STS patients are 24.1% and 6.1%, respectively, whereas DDLPS patients have MFS and LRFS of 14%-17%, respectively. Moreover, tumors with the same diagnosis but distinct anatomical sites exhibit variable results. For instance, compared to extremities DDLPS, retroperitoneal DDLPS exhibits a worse five-year LRFS (20% vs. 62%) [4,9].

Currently, the two most significant factors that physicians utilize to assess prognosis are tumor size and grade [10]. Numerous studies have shown that tumor size is a highly reliable positive predictor of both MFS and OS8. Tumors < 5 cm (at maximum dimension) and those > 5 cm are usually classified according to their different risk levels, with patients belonging to the lower and higher risk groups. Nonetheless, cellular differentiation status, mitotic count, and the degree of necrosis present are all integrated into the correlation between size and FNCLCC grading. There are constraints to this methodology; however, it is mostly used in selecting patients for adjuvant chemotherapy. Grading is not particularly beneficial for suspected high-grade confirmed sarcomas. Furthermore, grading in the limited diagnostic biopsy tissue is difficult due to intra-tumoral heterogeneity [11]. This frequently leads to an undergrading of STS, especially in LMS, and hence, prevents patients from receiving potentially helpful treatment [12].

Apart from traditional microscopy and electron microscopy, classical histopathology has expanded quickly in recent years with the development of other highly sensitive diagnostic techniques like immunohistochemistry, cytogenetics, and molecular pathology. These techniques offer more reproducible, reliable criteria for tumor diagnosis, classification, and follow-up. Immunohistochemistry is a crucial tool in contemporary diagnostic histopathology, providing valuable insights into tumor identification and the treatment of cancer patients. Over the past two decades, immunohistochemistry has undergone significant advancements, becoming a highly specialized molecular method that integrates the concepts of immunology, biochemistry, and histology. As a result, it has become an extremely powerful instrument in the daily practice of diagnostic histopathology. These days, there are thousands of monoclonal and polyclonal antibodies that have been optimized for particular cellular and extracellular structures.

A panel of antibodies is needed in most situations, with a second panel based on the wide lineage indicated by the expression of certain antigens in conjunction with the morphology. For every type of tumor, immunohistochemical markers that are useful in the paraffin section are discussed in detail; nonetheless, some basic remarks are appropriate here. Since antibodies are rarely completely sensitive or specific, relying too much on one marker is not advised. Being familiar with the reactivity pattern of each antibody used can assist prevent errors in diagnosis [4,5]. Additionally, the margins of small needle biopsy cores can display artifactually positive immunostaining. It is imperative to utilize a panel of antibodies and consider both positive and negative results in conjunction with the morphologic findings and clinical picture. The initial panel will be determined by the clinical situation and the morphologic category that the lesion belongs to. However, a first-line panel for spindle cell and pleomorphic sarcomas typically consists of desmin, smooth muscle actin, S100 protein, a broad-spectrum cytokeratin, and CD34; for intraabdominal pleomorphic sarcomas, additional panels may include MDM2, CDK4, and p16. An initial panel of tests for epithelioid and clear cell soft tissue cancers may consist of cytokeratin, EMA, CD34, CD31, CD30, desmin, and S100 protein; in certain situations, INI1 may also be added. A helpful panel for small round cell cancers includes FLI-1, WT1, myogenin, TLE1, NB84a, synaptophysin, chromogranin, neurofilament, and neuron-specific enolase in addition to CK, desmin, CD99, S100 protein, CD56, CD45, and TdT. The proliferation index may be predictive for grade 2 or 3 sarcomas, in which case a proliferation marker like Ki-67 (MIB1) can be included [11,13].

Through the identification of particular cellular antigens on tissue sections obtained from frozen tissue, formalin-fixed paraffin-embedded tissue blocks, or even from cytology specimens, immunohistochemistry is crucial for determining the histogenetic origin of malignancies required for tumor categorization. It is also among the most effective ways to find less-than-ideal residual tumor cells in various areas, like bone marrow, lymph nodes, and surgical margins. This information is crucial for tumor staging and designing effective treatment plans. In addition, a multitude of significant prognostic and etiopathological markers are available through immunohistochemistry, which are intriguing for tumor research and surveillance. It is imperative to acknowledge that quantitative immunohistochemistry is a work in progress, and it is improbable that the prevalent cutoff-based prognostic immunohistochemistry used in most research publications today will play a significant role in precision medicine down the road [12,14].

## Materials and methods

This investigation was conducted as an observational cohort study at the Department of Orthopedics, Bucharest Emergency University Hospital, for a period of seven years from 2016 to 2023. The main goal was to detect and thereafter monitor patients who were diagnosed with STS in their extremities, namely in their upper or lower limbs. The study acquired permission from the local ethics council, and all subjects provided signed informed consent.

The study included patients who were 18 years of age or older and had a confirmed diagnosis of STS that affected either the upper or lower extremities. Patients with other malignancies were omitted to prevent any factors that could affect the outcomes associated with STS. Similarly, patients with incomplete or missing data on important research variables were also excluded.

The study gathered extensive patient data from hospital records, encompassing demographic information such as age and gender, tumor parameters including site, size, and depth, as well as histological data such as histologic type and grade established by the French Federation of Cancer Centers (FNCLCC) system. Complete tumor sections were analyzed to verify precise classification and type.

Diagnostic procedures encompassed imaging modalities such as MRI and CT scans, in conjunction with biopsies to definitively establish the diagnosis of STS. Immunohistochemistry, along with other advanced diagnostic techniques, was used to accurately classify the malignancies. The immunohistochemistry panel consisted of antibodies targeting desmin, smooth muscle actin, S100 protein, broad-spectrum cytokeratin, CD34, MDM2, CDK4, p16, and Ki-67 (MIB1).

Each patient was provided with a personalized treatment plan according to their unique clinical presentation. Patients diagnosed with localized STS underwent surgical procedures with the goal of achieving R0 resection margins. Limb-preserving procedures were prioritized in instances when total excision was difficult due to the size or location of the tumor. Preoperative radiotherapy was administered to reduce the size of big tumors before surgery, while postoperative radiotherapy was given to patients with R1 margins or other high-risk factors for local recurrence. Patients with metastatic disease were administered systemic chemotherapy either at the time of diagnosis or after surgery, according to the recommendations set by the European Society for Medical Oncology (ESMO). Treatment plans for chemotherapy were customized according to the specific histologic subtype of the cancer and the overall health condition of the patient.

Patients were carefully monitored for recurrence and survival. Subsequent visits comprised clinical examinations, imaging scans, and laboratory tests. The primary objectives of the study were OS and disease-free survival (DFS). The survival data were analyzed using Kaplan-Meier survival curves, and comparisons between groups were conducted using the log-rank test. Statistical analyses were conducted to assess the correlation between patient outcomes and other prognostic markers, such as tumor size, histologic grade, tumor location, presence of neurovascular or bone invasion, tumor necrosis, and adenopathy. Cox proportional hazards regression models were used to undertake multivariate analysis in order to uncover independent prognostic factors.

Immunohistochemistry was performed to evaluate the expression of particular markers. The study assessed the proliferation index, measured by Ki-67 (MIB1), to establish its association with tumor grade and patient survival. Additional indicators evaluated included desmin, smooth muscle actin, S100 protein, cytokeratin, CD34, MDM2, CDK4, and p16.

The study was carried out in compliance with the Declaration of Helsinki and received approval from the ethics committee of Bucharest Emergency University Hospital (approval no. 43121). Prior to being included in the study, all individuals gave their informed consent. The study was constrained by various limitations, such as a small sample size, which could potentially restrict the applicability of the findings. The observational design cannot show causation, and there is a possibility of selection bias due to the exclusion of patients with incomplete data. In addition, the diversity of treatment protocols may impact the capacity to compare outcomes.

## Results

**Figure 1:**
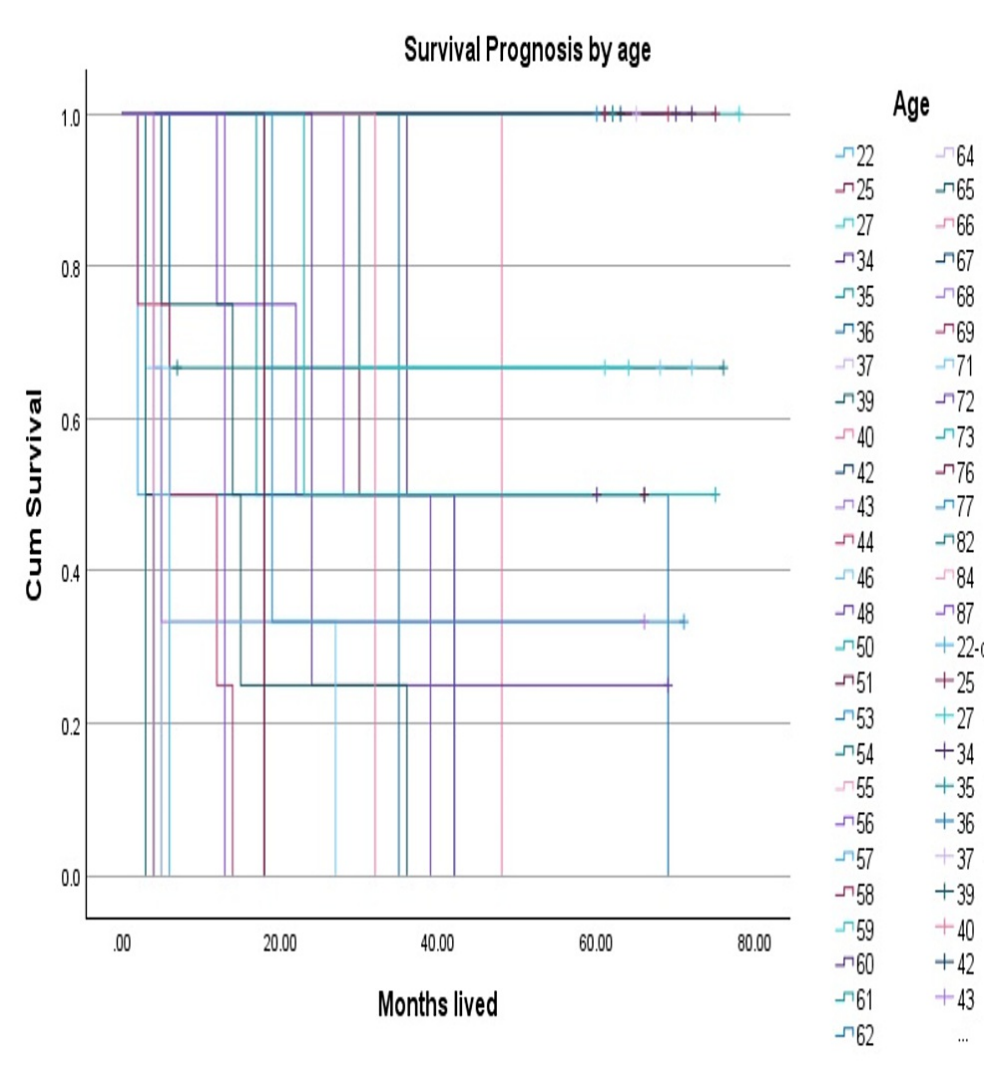
Survival prognosis by age

**Figure 2:**
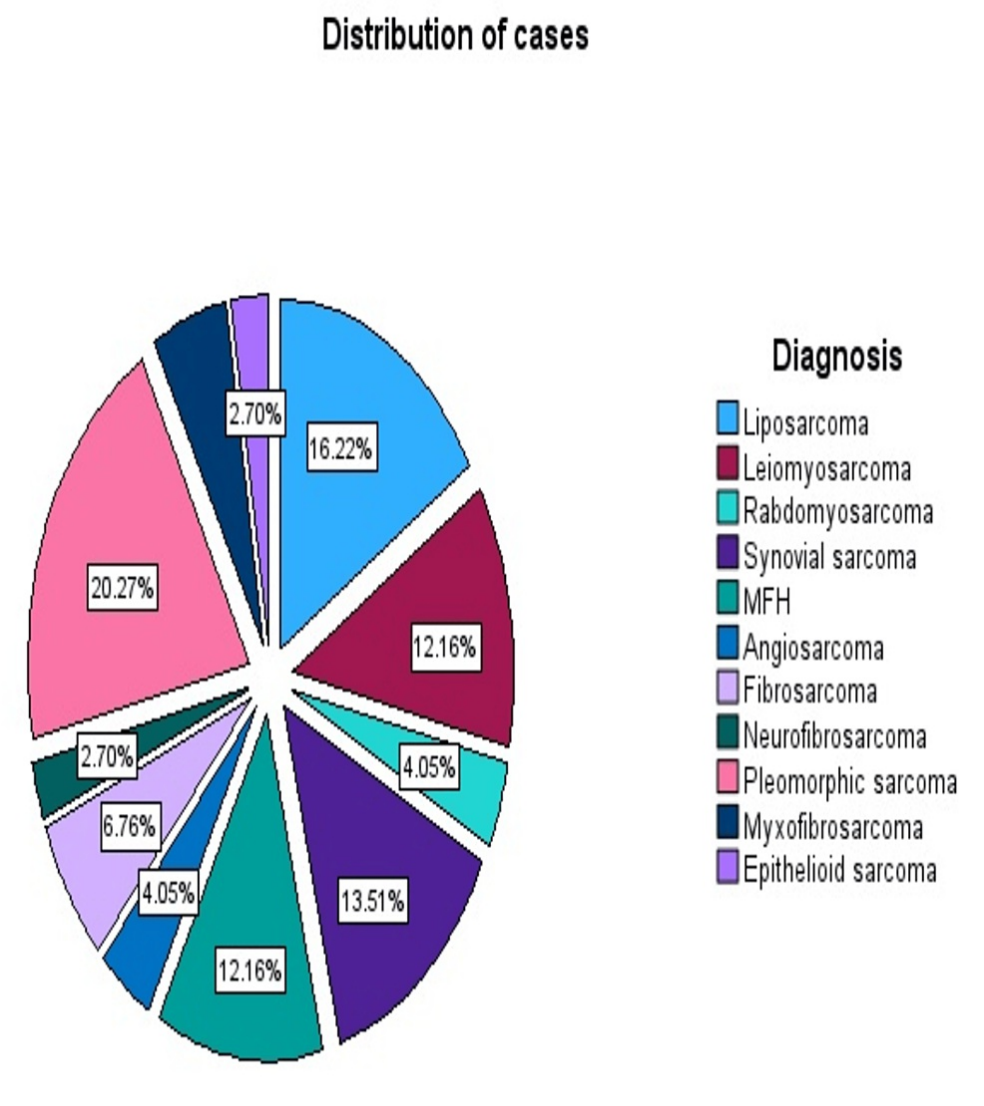
Distribution of cases by histotype

The median age at diagnosis was 53 years and 38 (52%) of the patients were women. Age was associated with higher mortality. The overall comparison of values between age and survival prognosis (Table 1, Figure 1) showed a significant correlation with a P-value of p<0.001 by applying the Kaplan-Meier survival curve. The most common histologic type encountered was pleomorphic sarcoma 15 (20.27%), followed by liposarcoma 12 (16.22%) and myxofibrosarcoma nine (12.16%) (Figure 2).

**Table 1:** Survival distribution for age

Overall Comparisons			
	Chi-Square	df	Sig.
Log Rank (Mantel-Cox)	104.348	39	< 0.001
Breslow (Generalized Wilcoxon)	90.215	39	< 0.001
Tarone-Ware	96.585	39	< 0.001
Test of equality of survival distributions for the different levels of age			

**Figure 3:**
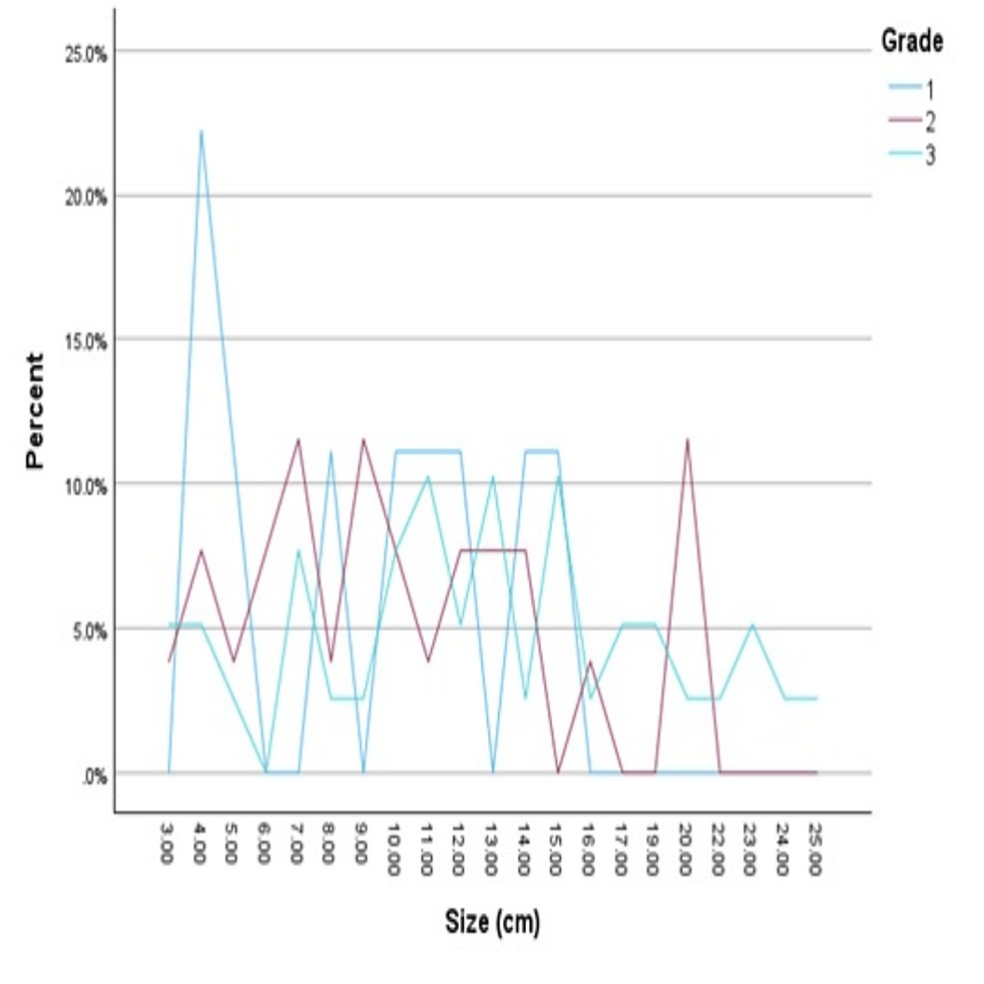
Correlation between size and grade

**Figure 4:**
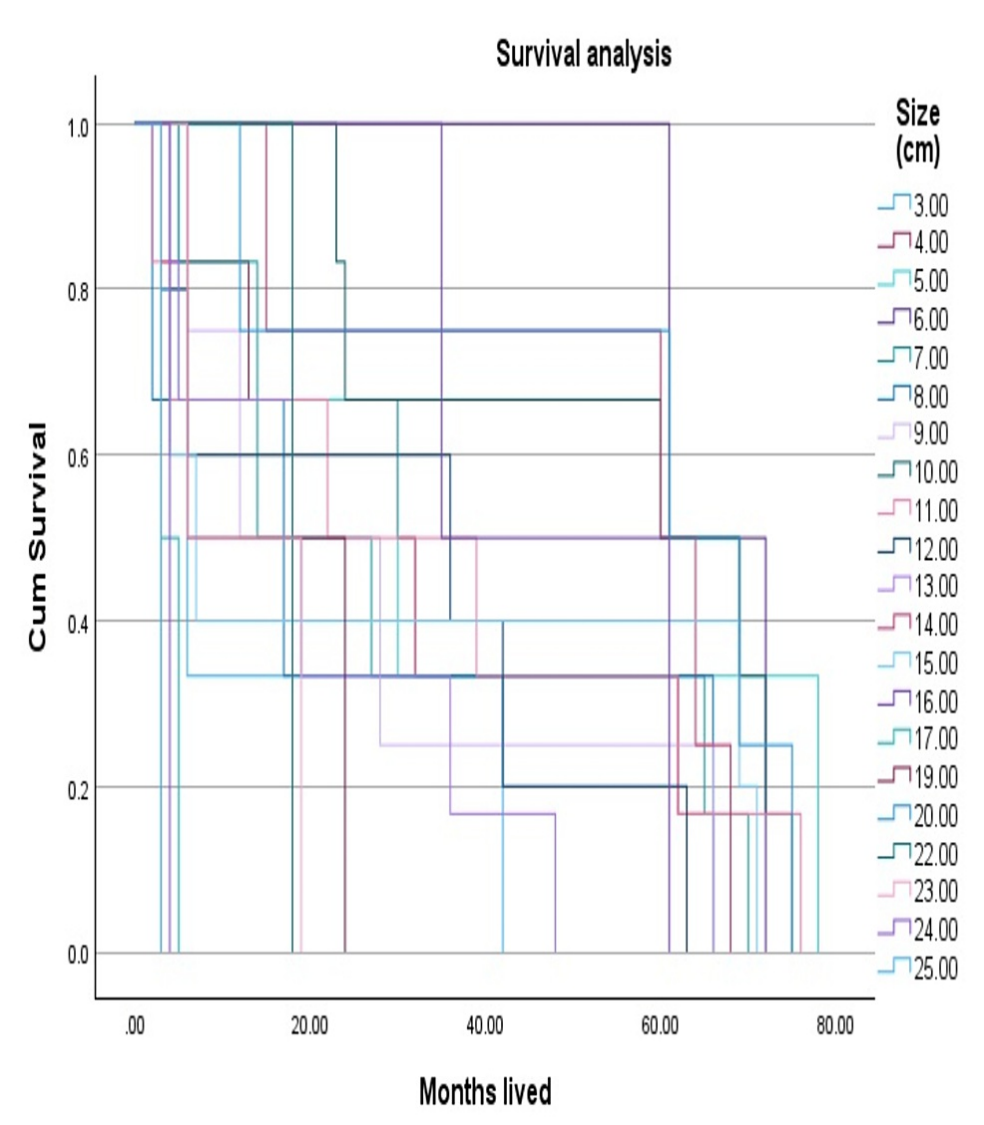
Overall survival analysis by size

Regarding the size of the tumor, 61 (83%) of the patients were diagnosed with a tumor exceeding 5 cm at the time of diagnosis. The OS study indicates that a poorer five-year survival rate and a higher-grade sarcoma are associated with size as shown in Figures 3, 4.

**Figure 5:**
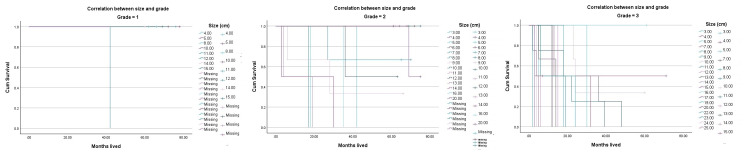
Correlation between grade and overall survival

The most significant predictor of outcome and the most accurate measure of the likelihood of metastasis in adult STSs is histologic grading. According to FNCLCC grading, in our study, 39 (52.70%) of the tumors were grade 3, 26 (35.14%) grade 2, and nine (12.16%) grade 1. Our findings revealed a shortened survival rate in patients diagnosed with grade 3 sarcomas, results shown in Figure 5.

**Figure 6:**
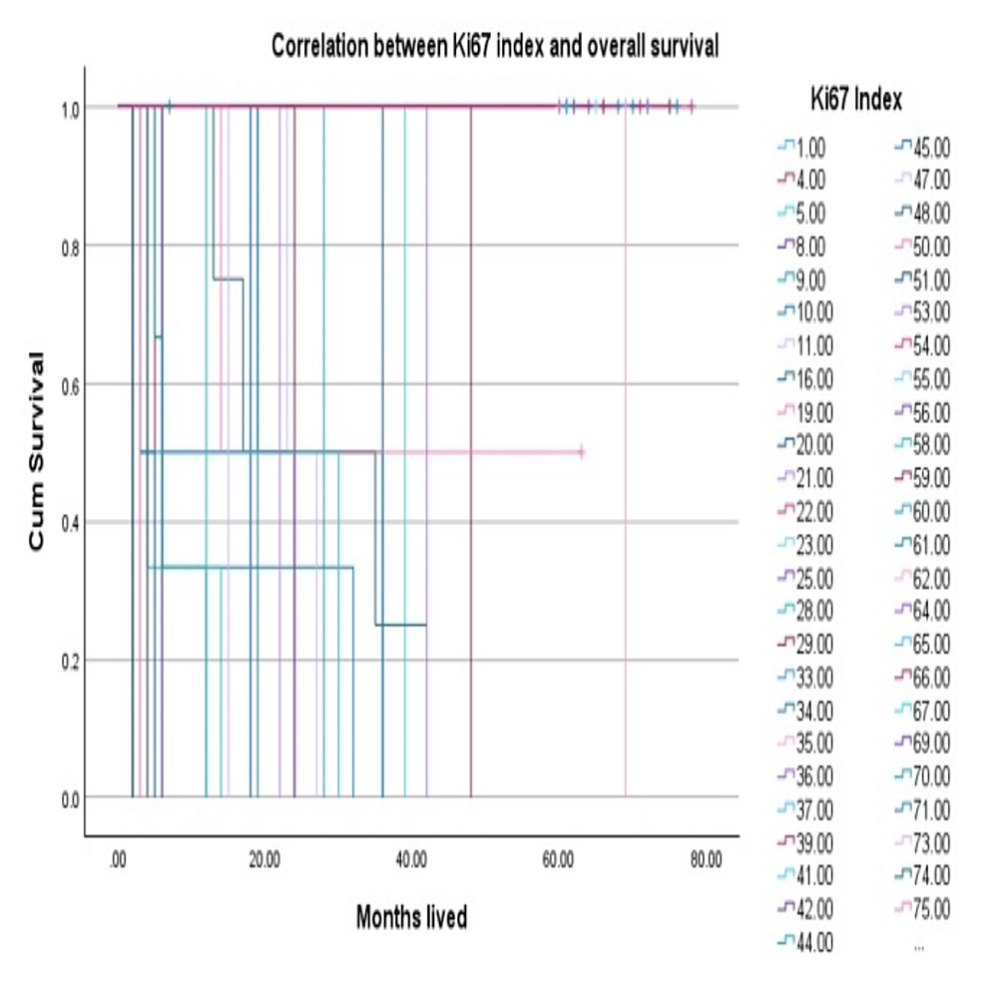
Correlation between Ki67 index and overall survival

The goal of the current study was to examine the significance of the Ki67 index in soft tissue tumors and determine whether OS and Ki67 analysis were correlated. The marker of proliferation (antigen Ki67) correlates with the prognosis of the patients and rate of survival as shown in Table 2 and Figure 6.

**Table 2:** Overall comparisons for Ki67 index and survival

Overall Comparisons			
	Chi-Square	df	Sig.
Log Rank (Mantel-Cox)	191.387	51	< .001
Breslow (Generalized Wilcoxon)	165.902	51	< .001
Tarone-Ware	177.413	51	< .001

Twenty (27%) of the patients were diagnosed with distant metastases at the time of admission into the hospital due to poor control of the tumor or incomplete oncologic treatment. The majority of lung metastases originate from original cancers with high grade. However, in a portion of the patients in this study, low-grade tumors progressed to lung metastases. These lesions have exhibited metastasis, which means that their biologic behavior is more in line with high-grade lesions.

**Figure 7:**
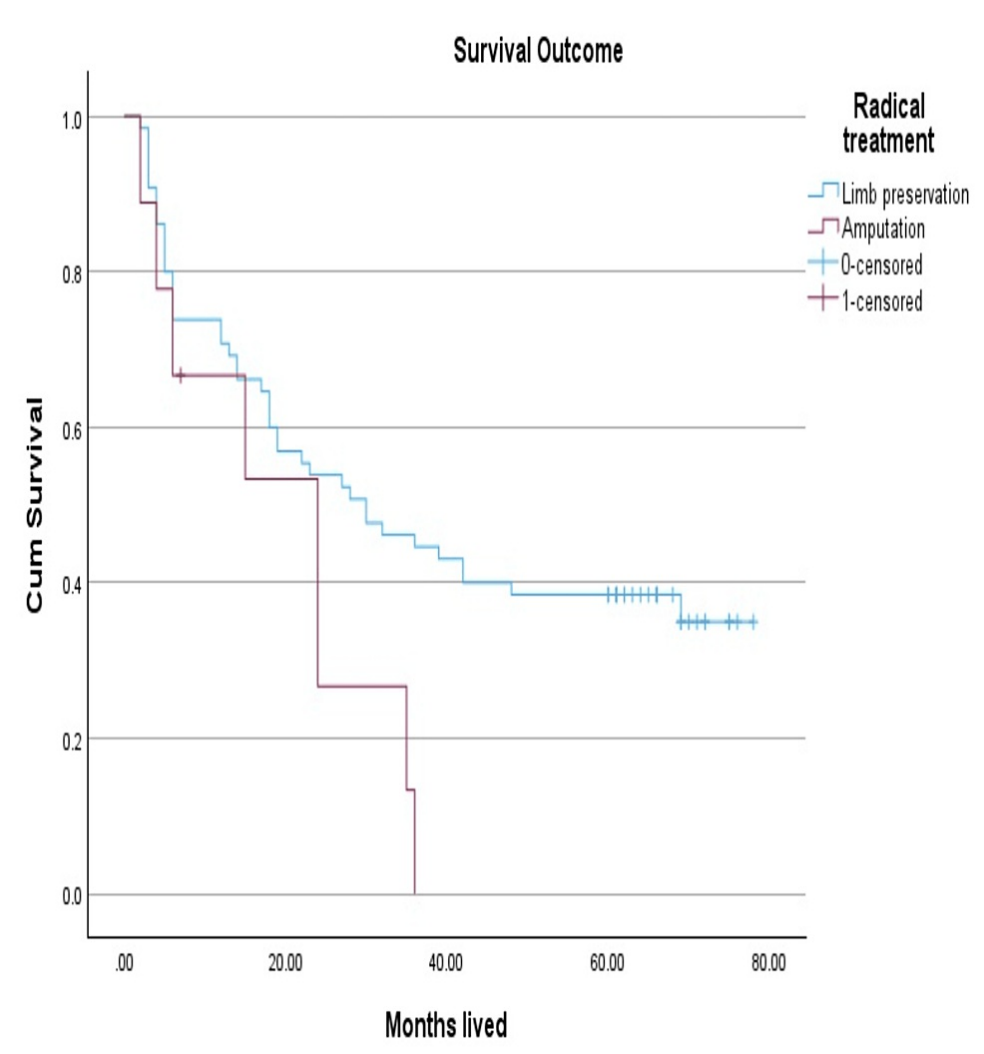
Survival analysis of patients with radical treatment

Approximately nine (12%) of our sarcoma cases underwent radical treatment (amputation of the affected limb or disarticulation). Patients who needed amputations either had tumors involving a critical neurovascular bundle or underlying bone, or they had neoplasms whose size would have required such a large resection that no functional limb would remain. Compared to published statistics on sarcoma resections generally, the prognosis of individuals who had their limbs amputated turned out to be less favorable in our study (Figure 7) [15].

Although the radical treatment by amputation or disarticulation in our study was used only in locally advanced cases or with invasion of the main vessels that did not achieve a reduction in the size of the tumor after chemotherapy or radiotherapy, so the results cannot have statistical value.

Prognosis is influenced by a variety of factors, including age, tumor size, histologic grade, depth (superficial or deep), histologic subtype, and site. Among the 74 patients, only 26 (35%) survived for more than five years with a median follow-up period of 7.5 years. In this case, early diagnosis, at an early stage of a low-grade STS, measuring under 5 cm correlated with OS and good functional outcome.

## Discussion

The main aim of this study was to improve the comprehension of prognostic variables in STSs and their implications for therapeutic therapy. The results of our study emphasize the crucial significance of histologic grading as the primary predictor of outcomes, specifically regarding the spread of cancer to other parts of the body and the total length of survival. This is congruent with the current body of literature, which constantly emphasizes that tumor size, depth, and grade are crucial factors for predicting outcomes.

The analysis of our data showed that a large proportion of 39 (52.70%) of the individuals in the study had grade 3 tumors. We also found a strong association between higher tumor grades and increased Ki67 proliferation index, as well as lower OS. This correlation was statistically significant (p<0.001). The results of this study support the effectiveness of the FNCLCC grading system in predicting outcomes and guiding treatment decisions. The study findings indicate that palliative chemotherapy has a significantly higher response rate in patients with high-grade sarcomas. This supports the rationale for utilizing chemotherapy as a strategic approach in the treatment of these aggressive malignancies [5,15].

Timely identification and treatment are essential for enhancing patient results. The results of our study showed that patients with early-stage, low-grade STS, especially those with tumors less than 5 cm, had higher survival rates and better functional outcomes. This highlights the importance of timely diagnostic and therapeutic procedures in specialist facilities to improve survival rates and minimize complications.

The study also emphasized the intricate and fluctuating nature of STS, which provides considerable difficulties in both diagnosing and treating the condition. Although immunohistochemistry and molecular pathology have made tremendous advancements in improving diagnostic accuracy and tumor categorization, there is still a need for more effective risk stratification tools to assist in clinical decision-making. A customized treatment approach is required due to the diversity within and among histologic subtypes, taking into account the unique characteristics of each patient and tumor [2,8,16].

In addition, our study found that a significant percentage of patients 20 (27%) had distant metastases at the time of diagnosis, primarily in high-grade tumors. This highlights the aggressive character of some STS subtypes and emphasizes the significance of early identification and extensive oncologic treatment. The correlation between larger tumors (>5 cm) and higher grades underscores the necessity for efficient monitoring and intervention methods since they are indicative of a more unfavorable prognosis.

Surgical procedures such as limb amputation or disarticulation were mostly performed on individuals with advanced illness or severe neurovascular complications. Although the interventions did not have a substantial impact on survival in our group, this is likely because the disease was already at an advanced stage when surgery was performed. However, it is important to emphasize the significance of personalized treatment strategies. The results indicate that aggressive surgery should be taken into account as part of comprehensive multimodal treatment strategies, particularly when the goal is to save the limb while still ensuring successful cancer treatment.

The study had several limitations. The limited sample size of 74 patients may restrict the capacity to apply the findings to a larger community of STS patients. Furthermore, due to the observational nature of the study, it is not possible to demonstrate causation, but simply relationships. Furthermore, there is a possibility of selection bias because patients with inadequate data were excluded. Moreover, the diversity of treatment protocols among individuals could impact the comparability of results. Ultimately, the study was carried out at a solitary site, perhaps constraining the generalizability of the findings to different environments or people. Although there are limitations, the study offers useful insights into the predictive characteristics of STSs and emphasizes the significance of personalized treatment options.

## Conclusions

This study emphasizes important prognostic indicators in STSs, such as tumor size above 5 cm, histological grade, and the specific kind of sarcoma. These factors have a substantial impact on the probability of distant metastasis and the survival of patients. The results indicate that severe interventions such as amputation or disarticulation do not necessarily enhance survival rates, especially in advanced stages. Accurate histological diagnosis, immunohistochemistry, and proper disease staging are crucial for the efficient management of sarcoma. This study emphasizes the significance of identifying high-risk patients for intensive treatment and low-risk patients to prevent unwanted side effects, with the ultimate goal of enhancing survival rates and quality of life for these individuals.
